# Supplementary material for: A New Era for Space Life Science: International Standards for Space Omics Processing
Source: Patterns (N Y). 2020 Nov 25;1(9):100148. doi: 10.1016/j.patter.2020.100148 (PMC7733874; doi:10.1016/j.patter.2020.100148)

PATTER, Volume *1*

Supplemental Information

A New Era for Space Life Science: International Standards for Space Omics Processing

Lindsay Rutter, Richard Barker, Daniela Bezdan, Henry Cope, Sylvain V. Costes, Lovorka Degoricija, Kathleen M. Fisch, Mariano I. Gabitto, Samrawit Gebre, Stefania Giacomello, Simon Gilroy, Stefan J. Green, Christopher E. Mason, Sigrid S. Reinsch, Nathaniel J. Szewczyk, Deanne M. Taylor, Jonathan M. Galazka, Raul Herranz, and Masafumi Muratani

Figure S1. Growth of GeneLab omics datasets by release year

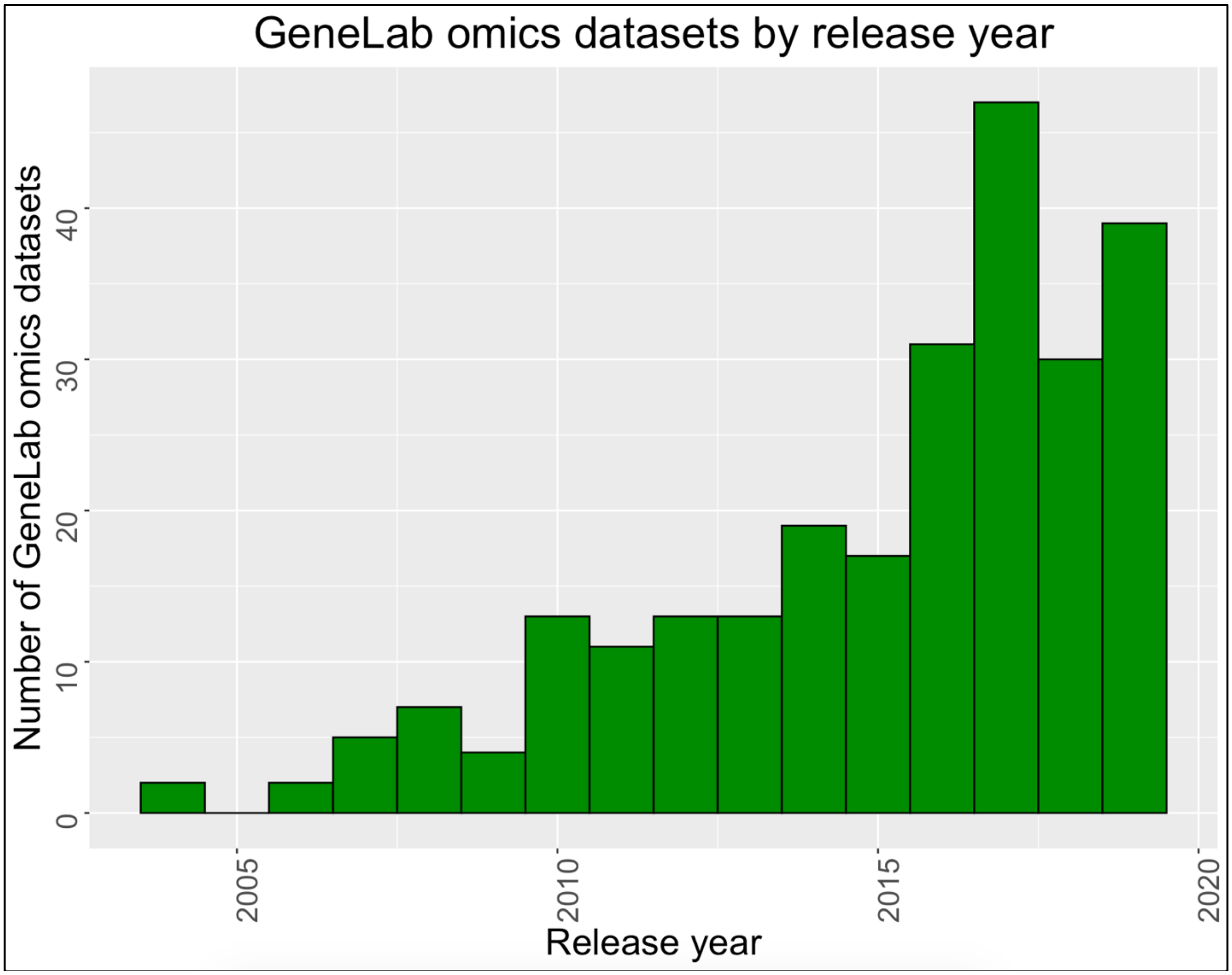

Figure S1. Growth of GeneLab omics datasets by release year

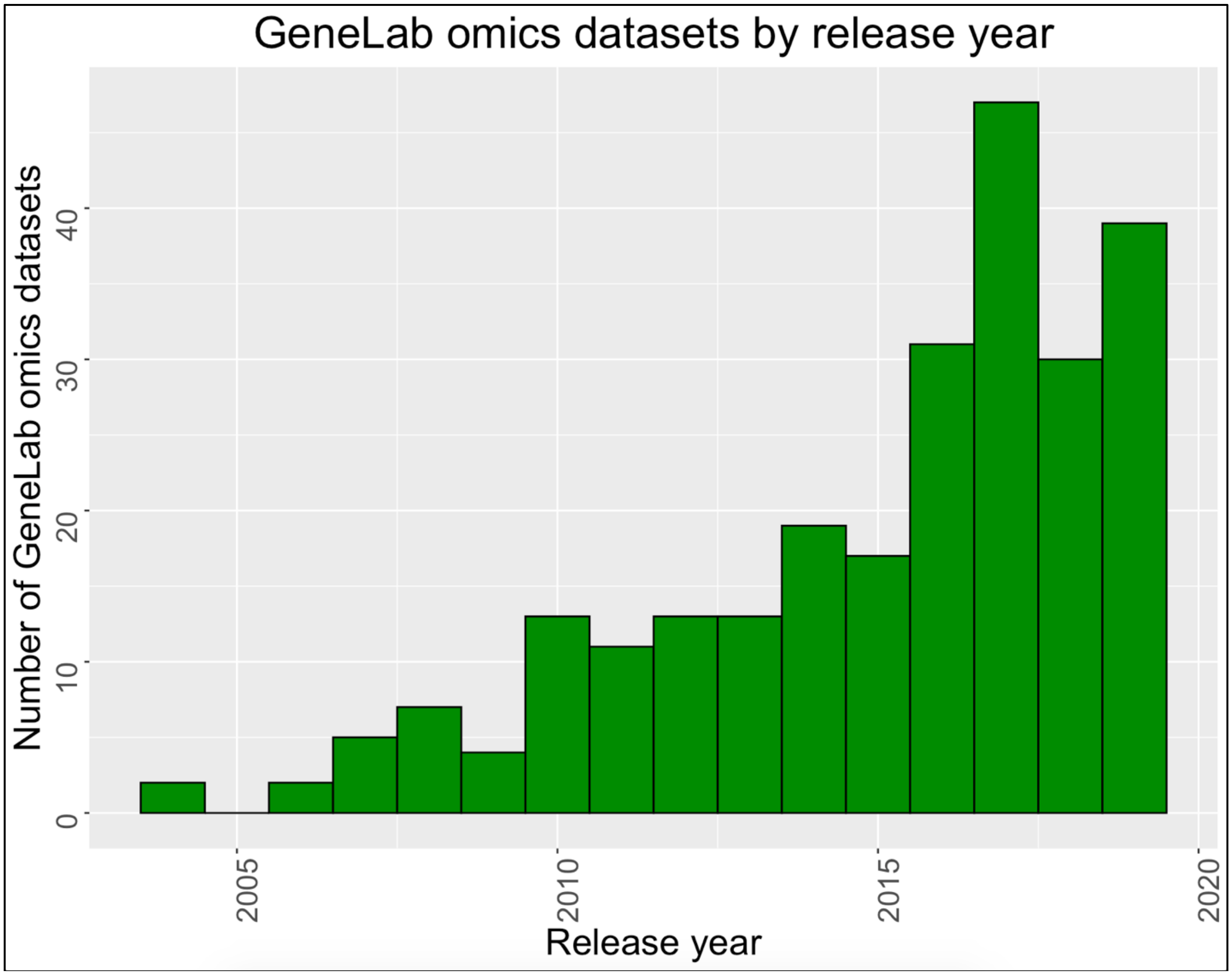

Supplement: Document S1. Figure S1 [file mmc1.pdf]
