# Supplementary material for: A New Era for Space Life Science: International Standards for Space Omics Processing
Source: Patterns (N Y). 2020 Nov 25;1(9):100148. doi: 10.1016/j.patter.2020.100148 (PMC7733874; doi:10.1016/j.patter.2020.100148)

Patterns

CellPress
OPEN ACCESS

Perspective

A New Era for Space Life Science: International Standards for Space Omics Processing

Lindsay Rutter,[1] Richard Barker,[2] Daniela Bezdan,[3] Henry Cope,[4] Sylvain V. Costes,[5] Lovorka Degoricija,[6] Kathleen M. Fisch,[7] Mariano I. Gabitto,[8] Samrawit Gebre,[6] Stefania Giacomello,[9] Simon Gilroy,[2] Stefan J. Green,[10] Christopher E. Mason,[11,12,13,14] Sigrid S. Reinsch,[5] Nathaniel J. Szewczyk,[15] Deanne M. Taylor,[16,17] Jonathan M. Galazka,[5,*] Raul Herranz,[18,*] and Masafumi Muratani[1,*]

[1]Transborder Medical Research Center and Department of Genome Biology, Faculty of Medicine, University of Tsukuba, Tsukuba, Ibaraki 305-8575, Japan
[2]Department of Botany, University of Wisconsin, Madison, WI 53706, USA
[3]Institute of Medical Virology and Epidemiology of Viral Diseases, University Hospital, Tubingen, Germany
[4]School of Computer Science, University of Nottingham, Nottingham NG8 1BB, UK
[5]Space Biosciences Division, NASA Ames Research Center, Moffett Field, CA 94035, USA
[6]KBR, NASA Ames Research Center, Moffett Field, CA 94035, USA
[7]Center for Computational Biology & Bioinformatics, Department of Medicine, University of California, San Diego, La Jolla, CA 92037, USA
[8]Flatiron Institute, Center for Computational Biology, Simons Foundation, New York, NY 10010, USA
[9]SciLifeLab, KTH Royal Institute of Technology, Stockholm 17165, Sweden
[10]Genome Research Core, University of Illinois at Chicago, Chicago, IL 60612, USA
[11]Department of Physiology and Biophysics, Weill Cornell Medicine, New York, NY 10065, USA
[12]The HRH Prince Alwaleed Bin Talal Bin Abdulaziz Alsaud Institute for Computational Biomedicine, Weill Cornell Medicine, New York, NY 10021, USA
[13]The WorldQuant Initiative for Quantitative Prediction, Weill Cornell Medicine, New York, NY 10065, USA
[14]The Feil Family Brain and Mind Research Institute, Weill Cornell Medicine, New York, NY 10065, USA
[15]Ohio Musculoskeletal and Neurological Institute (OMNI), Ohio University, Athens, OH 45701, USA
[16]Department of Biomedical and Health Informatics, The Children's Hospital of Philadelphia, Philadelphia, PA 19104, USA
[17]Department of Pediatrics, Perelman School of Medicine, University of Pennsylvania, Philadelphia, PA 19104, USA
[18]Centro de Investigaciones Biológicas "Margarita Salas" (CSIC), Ramiro de Maeztu 9, Madrid 28040, Spain
*Correspondence: jonathan.m.galazka@nasa.gov (J.M.G.), r.herranz@csic.es (R.H.), muratani@md.tsukuba.ac.jp (M.M.)
https://doi.org/10.1016/j.patter.2020.100148

THE BIGGER PICTURE With the rise of commercial spaceflight and prospective human missions to Mars, a wider health range of humans will enter space for longer spans and at higher exposure to environmental stressors than ever before. Numerous adverse health effects have been observed in space, including bone demineralization and skeletal muscle atrophy, among others. Scientists across the world are conducting space omics studies to develop countermeasures for safe and effective crewed space missions. However, optimal extraction of scientific insight from such data is contingent on improved standardization. In response, we founded ISSOP (International Standards for Space Omics Processing), an international consortium of scientists who aim to enhance guidelines between space biologists globally. This paper informs scientists and data scientists from many fields about the challenges and future avenues of space omics and can serve as an introductory reference for new members in the space biology discipline.

1 2 3 4 5 **Concept:** Basic principles of a new data science output observed and reported

SUMMARY

Space agencies have announced plans for human missions to the Moon to prepare for Mars. However, the space environment presents stressors that include radiation, microgravity, and isolation. Understanding how these factors affect biology is crucial for safe and effective crewed space exploration. There is a need to develop countermeasures, to adapt plants and microbes for nutrient sources and bioregenerative life support, and to limit pathogen infection. Scientists across the world are conducting space omics experiments on model organisms and, more recently, on humans. Optimal extraction of actionable scientific discoveries from these precious datasets will only occur at the collective level with improved standardization. To address this shortcoming, we established ISSOP (International Standards for Space Omics Processing), an international consortium of

This is an open access article under the CC BY license (http://creativecommons.org/licenses/by/4.0/).

scientists who aim to enhance standard guidelines between space biologists at a global level. Here we introduce our consortium and share past lessons learned and future challenges related to spaceflight omics.

BACKGROUND

Humankind has entered a new era of deep space exploration, with space agencies announcing plans to put humans back on the Moon in preparation for the first crewed missions to Mars. Radiation, microgravity, altered atmospheric gas composition, isolation, and diet changes are some of the known stressors on humans in the space environment; these factors are expected to increase with mission duration and distance outside of low Earth orbit.[1,2] Examples of adverse human health effects during spaceflight include bone demineralization,[3] skeletal muscle atrophy,[4] cardiovascular deconditioning,[5] vestibular control,[6] immune system suppression,[7,8] and neuro-ocular ailments.[9] It is necessary to better understand how spaceflight factors affect human health in order to develop the countermeasures needed for safe and effective crewed space missions. Moreover, critical elements of the space exploration infrastructure, including food and medical supplies, are insufficient for prolonged missions.[10]

The National Aeronautics and Space Administration (NASA) Twins Study further motivated the need for comprehensive, consortium-based approaches to study the long-term effects of spaceflight on humans.[11] Here, nine research groups studied a single data type in detail while a tenth group performed a multi-omics synthesis to construct a systematic whole-body layout of the changes. The study found alterations in numerous data types, including telomere length, gene regulation, gut microbiome composition, body weight, carotid artery dimensions, and serum metabolite profiles. While many of these changes were transient, some persisted for over 6 months after return to Earth.[11]

While the NASA Twins Study represents a step change in space biology research, it is also anomalous. The vast majority of space biology experiments and datasets are generated using model organisms (Figure 1). Animal models are used to infer how spaceflight affects humans; plant models are used to elicit how crops can be cultivated in space for food and renewed oxygen sources; and microbes are studied to understand how space affects human microbiomes, plant-microbe interactions, and environmental cleanliness, while also advancing the fields of space biotechnology, planetary protection, and astrobiology.[12,13] Specifically, the NASA Rodent Research (RR)[14] and Japan Aerospace Exploration Agency (JAXA) Mouse Habitat Unit (MHU)[15,16] series are part of a long heritage of rodent experiments in space; the zebrafish, medaka fish, fruit fly, and worm have all been valuable models for studying the effects of microgravity (μg), hypergravity, and space stressors using much larger sample size[17–24] and proper 1g controls in space via centrifuges and on ground via microgravity simulators;[25] plant models are consistently flown to investigate gravitropism[26] and now food production;[27] and microbial models have been guests on Apollo,[28] Space Lab 1,[29] the Space Shuttle,[30] and the International Space Station (ISS),[31] with recent interest turning toward understanding the natural microbiomes of spaceships[32,33] and astronauts.[34]

In all cases, space biologists around the world are increasingly reliant on omics approaches due to its ability to maximize the knowledge gained from rare spaceflight experiments (Figure S1). This includes epigenomics, transcriptomics, proteomics, metagenomics, and metabolomics.[11] While omics can generate vast quantities of data, potentially paving the way for successful space missions, optimal extraction of actionable scientific insights from these complex data will only occur with improved standardization and communication at the international level.

In recent years, various consortiums have formed to address the increasingly expensive, large, and complicated nature of biological data.[35–41] These committees implemented standards that significantly accelerated the scientific progress of their respective communities. The space omics community remains relatively new and can adopt successful frameworks from these exemplar groups. At the same time, guidelines developed by these groups cannot simply be replicated into the space omics discipline. Conducting biological research in spaceflight presents unique technical and biological challenges that have not been met before and will need to be specifically addressed by the international space biology community to ensure its success.

In response, we have formed a consortium called International Standards for Space Omics Processing (ISSOP). Our members are scientists who conduct space omics experiments funded by multiple space agencies in Japan (JAXA), Europe (including delegates from the European Space Agency [ESA] Space Omics Topical Team[42]), and the United States (NASA). We bring expertise related to the processing of space omics samples from humans, vertebrate, and invertebrate model organisms, plants, and microbes; the implementation of multi-omics and systems biology approaches in space biology; and the normalization of spaceflight metadata. We are also informed with the latest developments across government, industry, and academia. Our mission is to develop, share, and encourage sample-processing standardization and metadata normalization of spaceflight omics experiments to allow for a better harmonization of data and increased gain of knowledge.

In this paper, we begin by describing examples of past lessons learned from omics studies on model organisms in space. These examples showcase the unique technical and biological challenges inherent to performing spaceflight omics and underline the need for improved standardization in the discipline. We then announce the current formation of ISSOP to address these needs at the international level. We close with a brief section with potential future avenues for ISSOP to bring standardized and systematic science to the space omics field.

PAST LESSONS LEARNED FROM SPACE OMICS WITH MODEL ORGANISMS

There are a number of unique challenges during each stage of a space omics experiment. In this section, we summarize those challenges and any advances therein that have been

CellPress
OPEN ACCESS

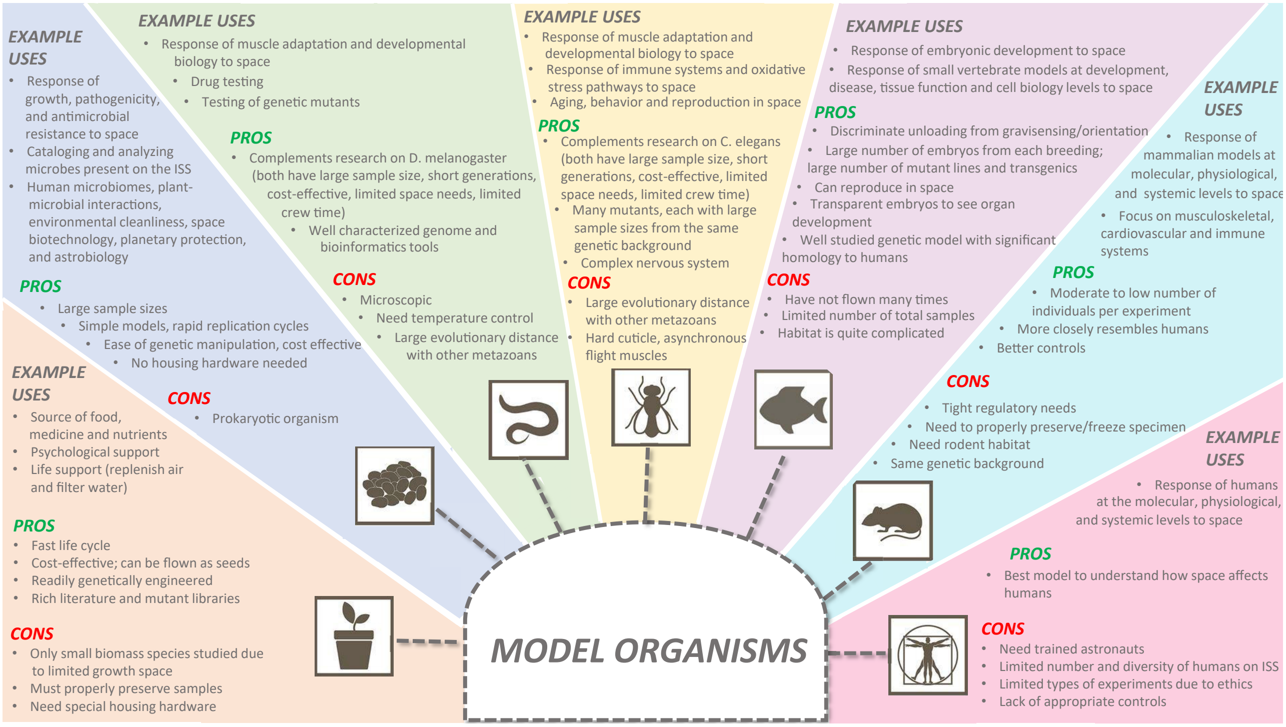

Figure 1. Example Uses, Pros, and Cons of Various Model Organisms Used in Space Omics Experiments

achieved in recent years through model organism studies. We journey through this section in roughly the same order of stages that researchers conduct space omics research projects (Figure 2).

Planning and Experimental Phase

Limitations in Space, Time, and Finances

The space omics community faces novel technical difficulties when planning spaceflight experiments. Basic challenges include logistical limitations in space, time, and finances. First, capacity limitations on orbiting platforms restrict the number of experimental replicates and variables, especially for rodent and plant studies. Small replicate numbers constrain statistical power and limit strain diversity. Genetically diverse crops will be crucial to foster robust bioregenerative life support systems in long-term space missions; however, the majority of plant species studied in space have been confined to low biomass species due to limited volume.[43] Second, crew time is exceptionally limited for experimental procedures in spaceflight. In 2019, an hour of astronaut time was valued at $17,500,[44] while the mean hourly wage in the United States for biochemists and biophysicists was $52.01.[45] These numbers roughly indicate it can be more than 300 times more expensive to perform experiments on orbit versus terrestrially. It is difficult to perform certain procedures on the ISS due to the small crew size and the lack of laboratory equipment and experience compared with what is common in terrestrial laboratories. Third, repeating unsuccessful experiments and following up successful experiments are both difficult due to logistical and financial constraints and typically face much longer waiting times compared with experiments on the ground.[43]

Hardware and Housing

Biological experiments during spaceflight are rarely performed using standard ground equipment. Developing special hardware and housing technology that can operate in spaceflight conditions is an ongoing challenge. In the past few decades, several space research platforms for animal and plant physiology have been developed.[46–49] Alongside these engineering advances, it has become clear that the hardware itself and how it is employed in experimental design must be carefully standardized and iteratively improved to mitigate unintentional confounding factors as they become better known.

For example, the standard rodent vivarium cages used in ground studies are unsuitable in microgravity. One hardware design that has proved to be an effective platform for rodent studies during spaceflight is the NASA Animal Enclosure Model (AEM).[49] A recent meta-study compared AEM ground controls and vivarium ground controls by examining all datasets in the NASA GeneLab database that had samples for both conditions.[50] The authors applied an unbiased systems biology approach that revealed substantial transcriptional differences in ground control rodents when only the habitat was changed.[50] In particular, a mild hypoxic phenotype was observed in the AEM condition, possibly due to its intentional design to intake ambient air, thereby passively intaking higher CO_2 concentrations implemented to match the spaceflight environment.[50] Importantly, increased CO_2 levels may cause a decrease in cognitive scores and an increase in headaches in humans.[51,52] Overall, this study underlined the critical need for well-designed ground control

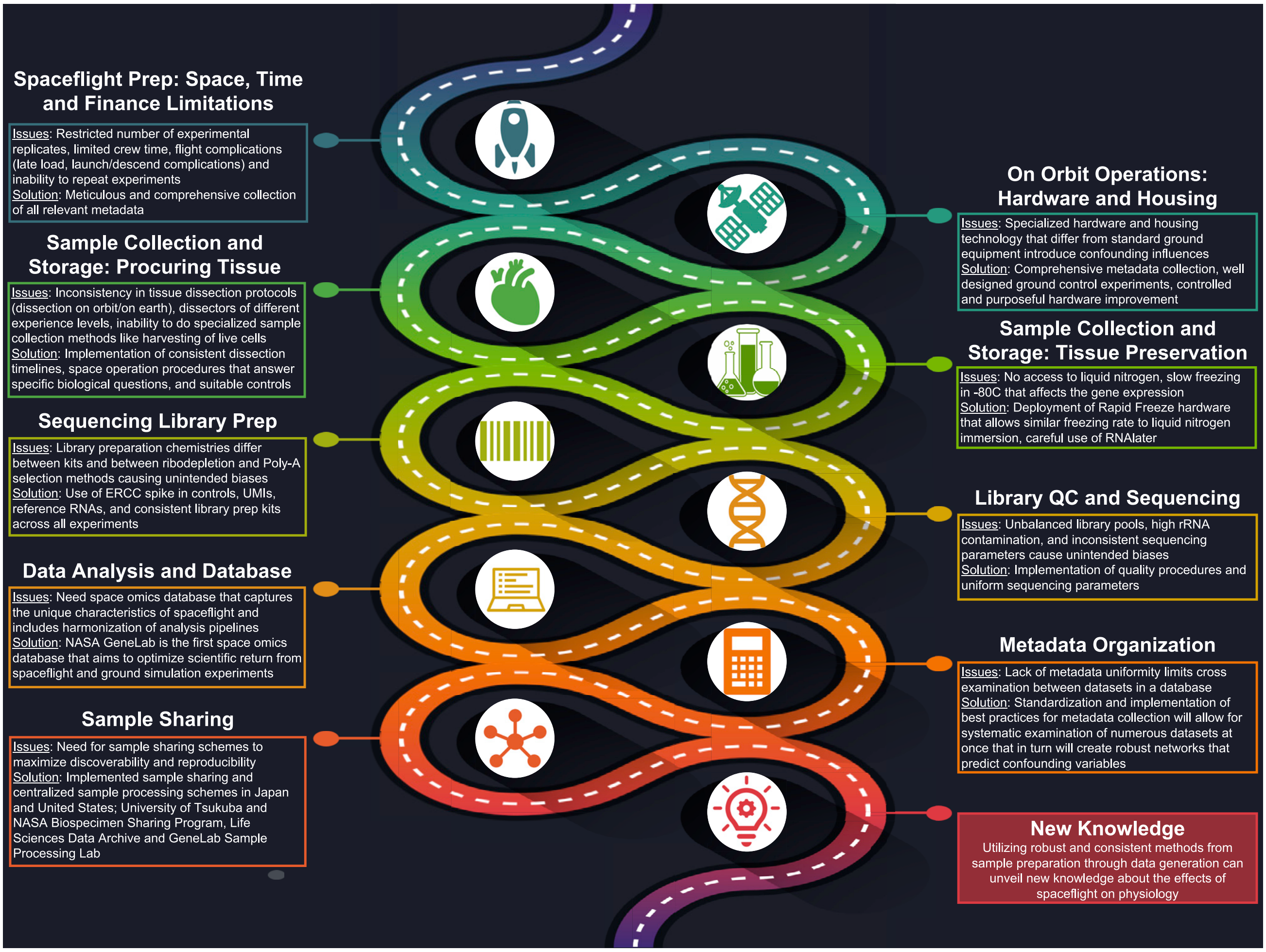

Figure 2. Roadmap of the Unique Challenges and Solutions during Each Stage of a Space Omics Experiment

experiments in order to address confounders that might otherwise lead to incorrect conclusions about the omics effects of spaceflight, something that has also been observed in flies cultivated in space.[25]

Plants also require special hardware designs for spaceflight, several of which have been used enough to understand that the hardware itself introduces extraneous variables. For example, Biological Research in Canisters (BRIC) hardware, which required no power and only limited crew time, was employed in numerous astrobotany experiments and found to have several shortcomings. The hardware itself reduced plant endodermal cell size, partially due to etiolated response to its dark environment.[53] Similarly, the BRIC Petri Dish Fixation Unit (BRIC-PDFU) hardware induces stress-related changes in the transcriptome and proteome of *Arabidopsis* seedlings, highlighting the continued need for iterative hardware revisions henceforth.[54]

The spaceflight community redevelops hardware not only to improve design but also to add new features that conserve time and effort for crew members. These features include real-time imaging, ground commanding, and automated software. New features are also added to better differentiate between the combinatorial effects of living in space. For example, in contrast to the NASA BRIC and BRIC-PDFU platforms, both the European KUBIK incubator and the JAXA Multiple Artificial-Gravity Research System (MARS) platforms provide 1*g* in-flights controls.[15] These controls may mitigate the risk of falsely identifying omics results as responsive to microgravity exposure when they are instead relevant to other spaceflight variables.

Nonetheless, we note that, as is the case with most innovative spacecraft housing units, these platforms themselves introduce their own confounding influences. In particular, gravity gradients appear across the rotor system and sample positions do not experience the same gravity force. Given that small differences in partial gravity exposure cause large transcriptional profiles changes in plants,[55,56] it is paramount for plant biologists who use these platforms to publish metadata that include both nominal partial *g* and real partial *g* for each sample position.

The use of reliable 1*g* controls both on Earth and in orbit is greatly acknowledged.[24,57,58] A large variety of ground-based simulation systems[55] can be used together to dissect the differential contribution of each confounding factor introduced by spaceflight or hardware requirements. Microgravity simulators include 2D clinostats, random positioning machines (RPMs), rotating wall vessels (RWVs), and diamagnetic levitation.[59]

Each of these simulators brings specific artifacts; for instance, clinostats introduce centrifugal accelerations and vibrations and diamagnetic levitation affects cell components differently based on magnetic susceptibility.[59] For these reasons, consistent terminology must be used and standards must be proposed for different simulators and different modes of operation.[59]

We note that commercial platforms are increasingly being contracted for spaceflight biological experiments, including TangoLabs (Space Tango, Lexington, KY), NanoLabs (NanoRacks, Houston, TX), and ICE Cubes Facilities (Space Application Services, Sint-Stevens-Woluwe, Belgium). To improve standardization and share lessons learned, communication will be increasingly crucial between the academic, government, and industry sectors developing and improving upon hardware designs. Consistent metadata collection about hardware used will also be crucial for informed interpretation of space omics data.

Sample Collection and Storage

Sample Collection

Due in part to limitations in crew time and finances, inconsistencies have arisen in how samples are obtained for analysis. For example, in some rodent experiments, animals are euthanized in space (''ISS terminal'') and either preserved as whole frozen carcasses (''ISS terminal frozen return'') for eventual dissection on Earth or dissected immediately by astronauts (''ISS terminal dissected return''). Alternatively, animals can be returned to Earth alive for euthanasia and dissection (''live animal return''). Each method has its strengths and weaknesses: While ISS-terminal approaches fix spaceflight responses directly, live animal return saves the time and specialized training astronauts would need to perform fine anatomical dissection on the ISS and ensure that these procedures are instead performed by professional technicians on Earth. Live animal return also allows scientists to study the offspring of returned spaceflight mice and to examine recovery to Earth conditions. On the downside, live animal return is confounded by exerted forces during reentry, reacclimation to Earth conditions after splashdown and before dissection, and circadian rhythm differences.

Sample Preservation

Adequate preservation of samples aboard the ISS is a continuing challenge and a hindrance to capturing unchanged biological responses to the orbital environment. Without access to liquid nitrogen, the current tissue freezing standard on the ISS is -80°C slow freezing. Unfortunately, NASA Rodent Research-1 (RR-1) missions revealed that space mouse gene expression analysis may not be reliable with slow freezing.[14] Indeed, slow freezing of mouse carcasses on orbit for eventual dissection on Earth (ISS terminal frozen return samples) showed large gene expression changes when compared with mice dissected by astronauts in orbit (ISS terminal dissected return samples). This appeared to be exacerbated by the use of poly(A) enrichment-based RNA sequencing (RNA-seq) protocol, and could be alleviated by the use of a ribodepletion-based approach and by snap-freezing carcasses in liquid nitrogen.[60] As a result, the Rapid Freeze hardware was recently developed for use on the ISS. The device can freeze mouse tissues (Glovebox freezer) and whole carcasses (Cryochiller) at rates similar to those attained with liquid nitrogen immersion. Studies are ongoing to determine how this new hardware compares with current ISS standard methods.

RNAlater consists of quaternary ammonium sulfates and cesium sulfates that denature and deactivate ribonucleases to prevent sample degradation. It is one fixative that has been widely used in space omics because it efficiently preserves nucleic acids and is deemed safe in the orbital environment. However, a secondary control study found that numerous genes identified in spaceflight experiments exhibit more pronounced differential expression induced by RNAlater than by microgravity.[61] The fixation process is not rapid enough to prevent a response to the preservative in plants or invertebrates. Under normal circumstances, parallel fixation of treatment and control samples would account for the preservation effects. However, spaceflight is by no means a normal circumstance: it is likely that the fixation process is markedly altered in microgravity, which induces sweeping alterations to structural components of plants and invertebrates and differences in fluid dynamics. Ultimately, transcriptomic experiments performed aboard the ISS should preferentially use snap-freezing preservation. In the absence of snap-freezing capabilities, experimentalists should examine the overlap in response to RNAlater and their experimental treatments to eliminate potential fixative-induced transcriptome events.[61,62]

Data Curation and Distribution

The Cancer Genome Atlas (TCGA) project significantly enhanced our understanding of complex biology with its carefully curated and publicly available multi-omics database.[36] In order to unravel how biology responds to space factors, the space omics community needs to capture this spirit and construct an analogous database tailored to the unique characteristics of space omics data. NASA GeneLab is the first comprehensive space omics database that aims to optimize scientific return from spaceflight and ground simulation experiments funded by multiple space agencies around the world.[63] The repository currently maintains more than 300 transcriptomic, epigenomic, proteomic, metabolomic, and metagenomic datasets from plant, animal, and microbial space experiments. Users can upload, download, store, and analyze spaceflight-relevant omics data in an open-access manner.

GeneLab complies with FAIR (findability, accessibility, interoperability, and reusability) principles[64,65] and houses raw, intermediate, and fully processed data files. This renders the data accessible to citizen scientists and scientists at every level. Users can fully reproduce each step of the analysis pipeline and reanalyze data using their preferred bioinformatics tools starting from any step in the pipeline. As raw and intermediate files lack interpretable biological meaning, processed data files contain user-friendly menus that allow users to easily explore statistical comparisons and visualizations between data in order to generate new space biology hypotheses at high and interconnected levels.[66]

The power of a database is greater than the sum of its individual datasets, but only if its individual datasets can be cross-examined in intelligent ways that assist with pattern discovery. Metadata are datasets that provide information about other datasets and hence are the bedrock of interconnecting datasets in informed manners. For this reason, implementation of best practices for metadata is just as important as for the datasets themselves.

GeneLab links critical metadata to each dataset. Metadata cover numerous factors that may present confounding variables during space omics experiments: biology factors (such as age, gender, strain, and ecotype), lifestyle factors (such as diet, exercise, and light cycle), experimental design factors (such as hardware and preflight and postflight exposure to stressors), sample-processing factors (such as preservation methods and library preparation methods), and spaceflight factors (such as gravity, atmospheric pressure, temperature, and ionizing radiation). Overall, metadata can be systematically examined to create robust networks that predict confounding variables and eventually determine additional experimental and engineering improvement areas for spaceflight omics studies.[67]

Metadata protocols continue to be refined in the space biology discipline. For example, ISS environmental metadata (such as CO_2, temperature, and radiation levels) are now integrated in space omics data.[68] Dosimeters are not typically incorporated in the housing units of space omics experiments; hence, dosage exposure for study samples must be extrapolated from surrounding dosimeters.[68] Meticulous metadata standardization efforts will continue to evolve and solve such challenges. External tools are also being developed to facilitate metadata reproducibility and discoverability. The Gilroy Astrobiology Team at the University of Wisconsin developed a metadata visualization API for the GeneLab platform called TOAST (Test of Arabidopsis Space Transcriptome) and a cross-species transcriptional viewer (NASA GeneLab Cross Kingdom Database) that uses iterative approaches to assist users with discovering common gene clusters among space omics datasets.[68]

As much as possible, space biologists employ standardized ontological vocabularies accepted by the larger scientific community through the Ontology for Biomedical Investigations (OBI). However, given the unprecedented nature of space biology, terminology must sometimes be extended from the OBI. This has been the case especially for radiobiology and space radiation terms. The addition of new ontology terms must be done carefully in manners that support controlled integration between datasets and metadata sources.[69]

Normalizing metadata is another enormous manual effort, sometimes requiring interviewing principal investigators and perusing publications to procure critical metadata. With growing volumes of incoming space omics datasets, this ambitious effort cannot be scaled.[69] Going forward, submission portals can be created to increase automatic curation, which has already been proved relatively successful in non-spaceflight applications.[69] Algorithms can guide data submitters to deposit crucial metadata and even provide explanations for why certain metadata are essential. Successfully relaying the importance of space omics confounders to researchers will improve their metadata submission adherence, thereby increasing the automation of metadata curation and the reliability of cross-data studies. Recent groups have indeed demonstrated the power of integrating multiple datasets from the GeneLab database to elicit global systemic responses to the space environment.[50] Future studies can similarly leverage the database and its thorough metadata to obtain the larger sample sizes and stronger statistical powers needed to further identify salient factors affecting space-flown organisms.

Sample Sharing

Deciphering fundamental molecular responses to space will likely not be achieved by a single research group. Sample-sharing schemes must be honed to maximize discoverability and reproducibility between researchers in the space omics discipline. To this end, sharing a common biobank and sample-processing facility is ideal. An organized, navigable biobank can allow researchers to determine whether tissues of interest are already available from previous studies and avoid conducting redundant, resource-consuming experiments in space. A central sample-processing facility can prevent batch effects that would otherwise be introduced in a multiple-facility configuration. The common facility can generate high-quality data using standard operating procedures (SOPs) performed by specially trained laboratory operators and robotic workstations. Overall, this framework would be in congruence with successful multi-omics endeavors, such as the TCGA project, where each type of omics was managed by a single center.[36]

Fortunately, space omics-sharing schemes are already implemented in Japan and the United States. A typical JAXA mouse live animal return study consists of 12 mice, producing more than 30 different tissue types across multiple omics assays that are then shared by more than 10 primary investigators. Genomics datasets from spaceflight mice are processed in a common laboratory at the University of Tsukuba that implements automated sample processing using LabDroids.[70] In the United States, unused frozen spaceflight samples from previous experiments are often archived in the NASA Biospecimen Sharing Program of the Life Sciences Data Archive. GeneLab scientists at the sample-processing laboratory process these samples and generate omics data using standardized methods for data reproducibility. While ESA does not have its own sample-sharing schemes, it does encourage multinational spaceflight experiments with sample sharing between European researchers and it does participate bilaterally with JAXA and NASA schemes. As the discipline forges onward, valuable sharing schemes for these rare and costly biological specimens returned from orbit should continue to be perfected.

CURRENT NEED FOR A GLOBAL CONSORTIUM: ISSOP

With an increasing reliance on and promise of omics technologies when properly standardized, and with different countries providing specific expertise, we have formed to create guidelines for space omics data with input from scientists at the global level. We envision that, as the field of human space omics matures, our guidelines can be readily extended from animal models to humans and to specifically allow for translatable inference and comparison between animal model data and human data. Our latest protocols will be available on our consortium website (https://issop.space) and GitHub (https://github.com/ISSOP) as they are developed and continuously updated.

Here we announce the inception of the ISSOP consortium. ISSOP is a portmanteau of the abbreviations ISS and SOP. We are an international consortium of scientists with a 3-fold mission statement. First, we develop, share, and encourage sample-processing standardization and metadata normalization of spaceflight omics experiments. Second, we aim to optimize the conditions for scientists and the general public to derive valid

Table 1. Objectives, Deliverables, and Expected Impacts of ISSOP

Community objectives	(1) Inform space biologists of recommended guidelines for space omics experiments; (2) Enhance information exchange between space biologists at the global level; (3) Reach a more diverse community of scientists and citizen scientists; (4) Incentivize engagement toward space omics and metadata standardization in the space biology research community; (5) Invite researchers committed to these values to join our efforts
Tangible deliverables	(1) Publish and routinely update our recommended sample-processing guidelines in a free and public repository; (2) Publish and routinely update lessons we have learned about space omics experiments in a free and public repository; (3) Upload key raw files, intermediate files, and final files using transparent and standardized data analysis pipelines for each dataset in the NASA GeneLab database; (4) Implement critical metadata standards for all datasets in the NASA GeneLab database
Shorter-term expected impacts	(1) Reduction in confounding factors and promotion of harmonization and interoperability between space omics datasets; (2) Increased accuracy when comparing between data, including historic spaceflight data with recently generated data, spaceflight analogue data with actual spaceflight data, and model organism data with human data; (3) Prevention of space biology researchers making the same expensive mistakes that have already been learned from other researchers; (4) Democratized access to priceless space omics data in various file formats accessible to both citizen scientists and seasoned bioinformaticians alike; (5) Accelerated derivation of valid hypotheses and novel discoveries related to the effects of space conditions on biological organisms
Longer-term expected impacts	(1) Improvements in hardware and software technology for future space omics experiments; (2) Advances in biological technologies, therapeutics, and countermeasures to support life in space; (3) Advances in biological technologies and therapeutics applicable to improvements of life on Earth

hypotheses from these precious data by reducing confounding factors and increasing interoperability at the global level. Third, our standardization efforts are crucial for understanding the effects of spaceflight on biological organisms and preparing the international community toward developing safe and effective crewed space exploration beyond low Earth orbit. Our objectives, deliverables, and expected impacts are listed in Table 1.

ISSOP meets regularly virtually and at the American Society for Gravitational and Space Research (ASGSR) conference, where we can communicate latest standardization guidelines to and from various industry representatives. Our collaborative meetings are designed to identify mechanisms to increase the impact of future space omics experiments while sharing new data and analysis methods. We invite interested readers to contact ISSOP with inquiries or suggestions at issop@issop.space. Corresponding authors in the current paper can also be contacted with questions specific to each geographical region within ISSOP (R.H. for Europe, M.M. for Japan, and J.M.G. for the United States).

FUTURE AVENUES

In this paper, we discussed the challenges of performing omics experiments in spaceflight on model organisms. An international consortium of scientists who bring expertise in space omics studies across a breadth of assay types and model organisms can best advance the field in a multifaceted manner. In future work, ISSOP can develop space omics recommendations across individual assays, including proteomics, metabolomics, metagenomics, transcriptomics, and epigenomics. Guidelines can also be developed for lesser-known but promising molecular biology laboratory techniques. For instance, JAXA and NASA projects have recently started to utilize laser microdissection (LMD) and spatial transcriptomics respectively for data collection at the tissue-part-specific level as opposed to simply the tissue-specific level. ISSOP members who participate in these studies can develop standardization protocols in the context of space omics. Best practices can also be proposed for various organisms. For example, as mentioned previously, physical limitations on orbit restrict sample sizes in certain organisms, including plants. ISSOP members with expertise in astrobotany can provide guidance on standards to maximize information from small numbers of samples during spaceflight. Digitization of sample handling with advanced robotics will be one of the important components for future remote experiments and project sharing. In total, ISSOP can present diverse and balanced guidelines for conducting space omics experiments across a range of assay types and model organisms; these guidelines can include quantitative and qualitative information about data collection, data extraction, library preparation, quality control, sample preservation, and sequencing parameters. It may be possible to eventually consolidate this information into a protocol decision tree algorithm that can provide standardized recommendations to principal investigators based on their organisms and assays of interest.

The challenges delineated in this paper will be further intensified as we leap into an age of human space omics. Commercial spaceflight will induce a wider health range of humans entering space while future long-term deep space missions will expose humans to more intense environmental stressors for longer durations than ever before. Fine-tuning the space telemedicine field will be necessary for these ambitious frontiers and will best be achieved with the addition of omics as a standard measures program. Pioneering crewed missions to Mars will likely require international input due to complexities in the technology and cultural ethics of working with human subjects.[71] ISSOP may be uniquely positioned to leverage lessons learned thus far from model organisms to develop an informed framework early on that can maximize scientific discovery and minimize ethical problems for an upcoming era of human space omics. Compellingly, careful standardization of space omics data through ISSOP may pave the way for cell space atlases[72,73] and precision

spaceflight medicine[74–78] that will critically improve the safety of humans traveling through space.

Here we have introduced ISSOP as an international consortium that can prime researchers to extract as much actionable insight as possible from space omics data through improved standardization, altogether benefiting upcoming trailblazing space missions during this critical period. This paper is intended to inform scientists and data scientists across a wide spectrum of disciplines about the challenges and future directions of the exciting field of space omics. This paper can also serve as an introductory reference for students and new members in the space omics and larger space biology discipline. We invite interested readers to learn more about ISSOP through our webpage. Satellite ISSOP papers will follow with more detailed focuses on specific realms of standard space omics processing, all intended to improve our understanding of the omics effects of spaceflight so humanity can reach new worlds safely.

SUPPLEMENTAL INFORMATION

Supplemental Information can be found online at https://doi.org/10.1016/j.patter.2020.100148.

ACKNOWLEDGMENTS

We thank Colin P.S. Kruse at Los Alamos National Laboratory for contributions about astrobotany challenges, Valery Boyko at NASA GeneLab for project conceptualization and figure creation, and Amanda M. Saravia-Butler at NASA GeneLab for contributions about sample preservation challenges. European (D.B., H.C., N.J.S., R.H., and S. Giacomello) contribution is supported by ESA Topical Team “Space Omics: Towards an integrated ESA/NASA –omics database for spaceflight and ground facilities experiments” grant 4000131202/20/NL/PG/pt to R.H. S. Giacomello is supported by Formas grant 2017-01066_3. H.C. is supported by the Horizon Centre for Doctoral Training at the University of Nottingham (UKRI grant no. EP/S023305/1) and by the NASA GeneLab Animal Analysis Working Group. N.J.S. is supported by the National Aeronautics and Space Administration (NNX15AL16G). NASA GeneLab members (J.M.G., S.V.C., S.S.R., L.D., S. Gebre) are supported by the NASA Space Biology program within the NASA Science Mission Directorate’s (SMD) Biological and Physical Sciences (BPS) Division. R.B. and S. Gilroy are supported by NASA (80NSSC19K0132). L.R. and M.M. represent the Omics Subgroup of Japan Society for the Promotion of Science (JSPS) KAKENHI funding group Living in Space and are supported by JP15K21745, JP15H05940, and JP20H03234. L.R. is supported by JSPS postdoctoral fellowship P20382. D.T. is supported by the Department of Biomedical and Health Informatics and The Children’s Hospital of Philadelphia Research Institute. K.F. is supported by the UC San Diego Department of Medicine and National Institutes of Health, grant UL1TR001442 of CTSA (Clinical and Translational Science Awards). C.E.M. is funded from the WorldQuant Foundation, The Pershing Square Sohn Cancer Research Alliance, and the National Institutes of Health (R01MH117406). S.V.C., J.M.G., and S.S.R. are supported by the GeneLab Project at NASA Ames Research Center, through NASA’s BPS division in the SMD; any use of trade names is for descriptive purposes only and does not imply endorsement by the US Government. C.E.M. is a co-Founder of Onegevity Health; D.B. is co-Founder of Analog LLC and Poppy Health Inc.; M.M. is an employee of LSI Medience Corporation through cross-appointment system of University of Tsukuba and Research Advisor of Robotic Biology Institute Inc.; and S. Giacomello is a scientific advisor of 10x Genomics Inc.

REFERENCES

1. Furukawa, S., Nagamatsu, A., Nenoi, M., Fujimori, A., Kakinuma, S., Katsube, T., Wang, B., Tsuruoka, C., Shirai, T., Nakamura, A.J., et al. (2020). Space radiation biology for “living in space”. Biomed. Res. Int. *2020*, 4703286.

2. Institute of Medicine (2008). Review of NASA’s Human Research Program Evidence Books (The National Academies Press). https://doi.org/10.17226/12261.

3. LeBlanc, A., Shackelford, L., and Schneider, V. (1998). Future human bone research in space. Bone *22*, 113S–116S.

4. Vandenburgh, H., Chromiak, J., Shansky, J., Del Tatto, M., and Lemaire, J. (1999). Space travel directly induces skeletal muscle atrophy. FASEB J. *13*, 1031–1038.

5. Bungo, M.W., Charles, J.B., and Johnson, P.C., Jr. (1985). Cardiovascular deconditioning during space flight and the use of saline as a countermeasure to orthostatic intolerance. Aviat. Space Environ. Med. *56*, 985–990.

6. Morita, H., Kaji, H., Ueta, Y., and Abe, C. (2020). Understanding vestibular-related physiological functions could provide clues on adapting to a new gravitational environment. J. Physiol. Sci. *70*, 17.

7. Konstantinova, I.V. (1991). Immune resistance of man in space flights. Acta Astronaut. *23*, 123–127.

8. Akiyama, T., Horie, K., Hinoi, E., Hiraiwa, M., Kato, A., Maekawa, Y., Takahashi, A., and Furukawa, S. (2020). How does spaceflight affect the acquired immune system? NPJ Microgravity *6*, 14.

9. Lee, A.G., Mader, T.H., Gibson, C.R., Brunstetter, T.J., and Tarver, W.J. (2018). Space flight-associated neuro-ocular syndrome (SANS). Eye (Lond) *32*, 1164–1167.

10. Douglas, G.L., Zwart, S.R., and Smith, S.M. (2020). Space food for thought: challenges and considerations for food and nutrition on exploration missions. J. Nutr. *150*, 2242, https://doi.org/10.1093/jn/nxaa188.

11. Garrett-Bakelman, F.E., Darshi, M., Green, S.J., Gur, R.C., Lin, L., Macias, B.R., McKenna, M.J., Meydan, C., Mishra, T., Nasrini, J., et al. (2019). The NASA Twins Study: a multidimensional analysis of a year-long human spaceflight. Science *364*, https://doi.org/10.1126/science.aau8650.

12. Ott, M., Pierson, D., Shirakawa, M., Tanigaki, F., Hida, M., Yamazaki, T., Shimazu, T., and Ishioka, N. (2014). Space habitation and microbiology: status and roadmap of space agencies. Microbes Environ. *29*, 239–242.

13. Yamaguchi, N., Roberts, M., Castro, S., Oubre, C., Makimura, K., Leys, N., Grohmann, E., Sugita, T., Ichijo, T., and Nasu, M. (2014). Microbial monitoring of crewed habitats in space-current status and future perspectives. Microbes Environ. *29*, 250–260.

14. Choi, S.Y., Saravia-Butler, A., Shirazi-Fard, Y., Leveson-Gower, D., Stodieck, L.S., Cadena, S.M., Beegle, J., Solis, S., Ronca, A., and Globus, R.K. (2020). Validation of a new rodent experimental system to investigate consequences of long duration space habitation. Sci. Rep. *10*, 2336.

15. Shiba, D., Mizuno, H., Yumoto, A., Shimomura, M., Kobayashi, H., Morita, H., Shimbo, M., Hamada, M., Kudo, T., Shinohara, M., et al. (2017). Development of new experimental platform “MARS”-Multiple Artificial-gravity Research System-to elucidate the impacts of micro/partial gravity on mice. Sci. Rep. *7*, 10837.

16. Matsuda, C., Kato, T., Inoue-Suzuki, S., Kikuchi, J., Ohta, T., Kagawa, M., Hattori, M., Kobayashi, H., Shiba, D., Shirakawa, M., et al. (2019). Dietary intervention of mice using an improved Multiple Artificial-gravity Research System (MARS) under artificial 1. NPJ Microgravity *5*, 16.

17. Hateley, S., Hosamani, R., Bhardwaj, S.R., Pachter, L., and Bhattacharya, S. (2016). Transcriptomic response of Drosophila melanogaster pupae developed in hypergravity. Genomics *108*, 158–167.

18. Hosamani, R., Leib, R., Bhardwaj, S.R., Adams, C.M., and Bhattacharya, S. (2016). Elucidating the “gravome”: quantitative proteomic profiling of the response to chronic hypergravity in Drosophila. J. Proteome Res. *15*, 4165–4175.

19. Ikenaga, M., Yoshikawa, I., Kojo, M., Ayaki, T., Ryo, H., Ishizaki, K., Kato, T., Yamamoto, H., and Hara, R. (1997). Mutations induced in Drosophila during space flight. Biol. Sci. Space *11*, 346–350.

20. Marcu, O., Lera, M.P., Sanchez, M.E., Levic, E., Higgins, L.A., Shmygelska, A., Fahlen, T.F., Nichol, H., and Bhattacharya, S. (2011). Innate immune responses of Drosophila melanogaster are altered by spaceflight. PLoS One *6*, e15361.

21. Ogneva, I.V., Belyakin, S.N., and Sarantseva, S.V. (2016). The development of Drosophila Melanogaster under different duration space flight and subsequent adaptation to earth gravity. PLoS One *11*, e0166885.

CellPress
OPEN ACCESS

22. Chatani, M., Mantoku, A., Takeyama, K., Abduweli, D., Sugamori, Y., Aoki, K., Ohya, K., Suzuki, H., Uchida, S., Sakimura, T., et al. (2015). Microgravity promotes osteoclast activity in medaka fish reared at the international space station. Sci. Rep. *5*, 14172.

23. Murata, Y., Yasuda, T., Watanabe-Asaka, T., Oda, S., Mantoku, A., Takeyama, K., Chatani, M., Kudo, A., Uchida, S., Suzuki, H., et al. (2015). Histological and transcriptomic analysis of adult Japanese medaka sampled onboard the international space station. PLoS One *10*, e0138799.

24. Higashibata, A., Hashizume, T., Nemoto, K., Higashitani, N., Etheridge, T., Mori, C., Harada, S., Sugimoto, T., Szewczyk, N.J., Baba, S.A., et al. (2016). Microgravity elicits reproducible alterations in cytoskeletal and metabolic gene and protein expression in space-flown. NPJ Microgravity *2*, 15022.

25. Herranz, R., Benguría, A., Laván, D.A., López-Vidriero, I., Gasset, G., Javier Medina, F., van Loon, J.J., and Marco, R. (2010). Spaceflight-related suboptimal conditions can accentuate the altered gravity response of Drosophila transcriptome. Mol. Ecol. *19*, 4255–4264.

26. Vandenbrink, J.P., and Kiss, J.Z. (2019). Plant responses to gravity. Semin. Cell Dev. Biol. *92*, 122–125.

27. Khodadad, C.L.M., Hummerick, M.E., Spencer, L.E., Dixit, A.R., Richards, J.T., Romeyn, M.W., Smith, T.M., Wheeler, R.M., and Massa, G.D. (2020). Microbiological and nutritional analysis of lettuce crops grown on the international space station. Front. Plant Sci. *11*, 199.

28. Berry, D., and Volz, P.A. (1979). Phosphate uptake in Saccharomyces cerevisiae Hansen wild type and phenotypes exposed to space flight irradiation. Appl. Environ. Microbiol. *38*, 751–753.

29. Sulzman, F.M., Ellman, D., Fuller, C.A., Moore-Ede, M.C., and Wassmer, G. (1984). Neurospora circadian rhythms in space: a reexamination of the endogenous-exogenous question. Science *225*, 232–234.

30. Crabbé, A., Nielsen-Preiss, S.M., Woolley, C.M., Barrila, J., Buchanan, K., McCracken, J., Inglis, D.O., Searles, S.C., Nelman-Gonzalez, M.A., Ott, C.M., et al. (2013). Spaceflight enhances cell aggregation and random budding in Candida albicans. PLoS One *8*, e80677.

31. Morrison, M.D., Fajardo-Cavazos, P., and Nicholson, W.L. (2019). Comparison of transcriptome profiles from two separate missions to the International Space Station. NPJ Microgravity *5*, 1.

32. Singh, N.K., Wood, J.M., Karouia, F., and Venkateswaran, K. (2018). Succession and persistence of microbial communities and antimicrobial resistance genes associated with International Space Station environmental surfaces. Microbiome *6*, 204.

33. Singh, N.K., Bezdan, D., Checinska Sielaff, A., Wheeler, K., Mason, C.E., and Venkateswaran, K. (2018). Multi-drug resistant Enterobacter bugandensis species isolated from the International Space Station and comparative genomic analyses with human pathogenic strains. BMC Microbiol. *18*, 175.

34. Voorhies, A.A., Mark Ott, C., Mehta, S., Pierson, D.L., Crucian, B.E., Feiveson, A., Oubre, C.M., Torralba, M., Moncera, K., Zhang, Y., et al. (2019). Study of the impact of long-duration space missions at the International Space Station on the astronaut microbiome. Sci. Rep. *9*, 9911.

35. MetaSUB International Consortium (2016). The metagenomics and Metadesign of the Subways and Urban Biomes (MetaSUB) international consortium inaugural meeting report. Microbiome *4*, 24.

36. Tomczak, K., Czerwińska, P., and Wiznerowicz, M. (2015). The Cancer Genome Atlas (TCGA): an immeasurable source of knowledge. Contemp. Oncol. *19*, A68–A77.

37. Ainsztein, A.M., Brooks, P.J., Dugan, V.G., Ganguly, A., Guo, M., Howcroft, T.K., Kelley, C.A., Kuo, L.S., Labosky, P.A., Lenzi, R., et al. (2015). The NIH extracellular RNA communication consortium. J. Extracell. Vesicles *4*, 27493.

38. Bernstein, B.E., Stamatoyannopoulos, J.A., Costello, J.F., Ren, B., Milosavljevic, A., Meissner, A., Kellis, M., Marra, M.A., Beaudet, A.L., Ecker, J.R., et al. (2010). The NIH roadmap epigenomics mapping consortium. Nat. Biotechnol. *28*, 1045–1048.

39. Carithers, L.J., and Moore, H.M. (2015). The genotype-tissue expression (GTEx) project. Biopreservation Biobanking, 307–308, https://doi.org/10.1089/bio.2015.29031.hmm.

40. Landt, S.G., Marinov, G.K., Kundaje, A., Kheradpour, P., Pauli, F., Batzoglou, S., Bernstein, B.E., Bickel, P., Brown, J.B., Cayting, P., et al. (2012). ChIP-seq guidelines and practices of the ENCODE and modENCODE consortia. Genome Res. *22*, 1813–1831.

41. Dekker, J., Belmont, A.S., Guttman, M., Leshyk, V.O., Lis, J.T., Lomvardas, S., Mirny, L.A., O'Shea, C.C., Park, P.J., Ren, B., et al. (2017). The 4D nucleome project. Nature *549*, 219–226.

42. Madrigal, P., Gabel, A., Villacampa, A., Manzano, A., Deane, C.S., Bezdan, D., Carnero-Diaz, E., Medina, F.J., Hardiman, G., Grosse, I., et al. (2020). Revamping Space-omics in Europe. Cell Syst. *11*. Published online November 25, 2020. https://doi.org/10.1016/j.cels.2020.10.006.

43. Vandenbrink, J.P., and Kiss, J.Z. (2016). Space, the final frontier: a critical review of recent experiments performed in microgravity. Plant Sci. *243*, 115–119.

44. Elburn, D. (2019). Commercial and Merkating Pricing Policy. https://www.nasa.gov/leo-economy/commercial-use/pricing-policy.

45. U.S. Bureau of Labor Statistics (2008). May 2019 National Occupational Employment and wage Estimates. https://www.bls.gov/oes/current/oes_nat.htm.

46. Cancedda, R., Liu, Y., Ruggiu, A., Tavella, S., Biticchi, R., Santucci, D., Schwartz, S., Ciparelli, P., Falcetti, G., Tenconi, C., et al. (2012). The Mice Drawer System (MDS) experiment and the space endurance record-breaking mice. PLoS One *7*, e32243.

47. Shimbo, M., Kudo, T., Hamada, M., Jeon, H., Imamura, Y., Asano, K., Okada, R., Tsunakawa, Y., Mizuno, S., Yagami, K., et al. (2016). Ground-based assessment of JAXA mouse habitat cage unit by mouse phenotypic studies. Exp. Anim. *65*, 175–187.

48. Beheshti, A., Shirazi-Fard, Y., Choi, S., Berrios, D., Gebre, S.G., Galazka, J.M., and Costes, S.V. (2019). Exploring the effects of spaceflight on mouse physiology using the open access NASA GeneLab platform. J. Vis. Exp. *143*, https://doi.org/10.3791/58447.

49. Moyer, E.L., Dumars, P.M., Sun, G.S., Martin, K.J., Heathcote, D.G., Boyle, R.D., and Skidmore, M.G. (2016). Evaluation of rodent spaceflight in the NASA animal enclosure module for an extended operational period (up to 35 days). NPJ Microgravity *2*, 16002.

50. Beheshti, A., Cekanaviciute, E., Smith, D.J., and Costes, S.V. (2018). Global transcriptomic analysis suggests carbon dioxide as an environmental stressor in spaceflight: a systems biology GeneLab case study. Sci. Rep. *8*, 4191.

51. Law, J., Van Baalen, M., Foy, M., Mason, S.S., Mendez, C., Wear, M.L., Meyers, V.E., and Alexander, D. (2014). Relationship between carbon dioxide levels and reported headaches on the international space station. J. Occup. Environ. Med. *56*, 477–483.

52. Allen, J.G., MacNaughton, P., Satish, U., Santanam, S., Vallarino, J., and Spengler, J.D. (2016). Associations of cognitive function scores with carbon dioxide, ventilation, and volatile organic compound exposures in office workers: a controlled exposure study of green and conventional office environments. Environ. Health Perspect. *124*, 805–812.

53. Johnson, C.M., Subramanian, A., Edelmann, R.E., and Kiss, J.Z. (2015). Morphometric analyses of petioles of seedlings grown in a spaceflight experiment. J. Plant Res. 1007–1016, https://doi.org/10.1007/s10265-015-0749-0.

54. Basu, P., Kruse, C.P.S., Luesse, D.R., and Wyatt, S.E. (2017). Growth in spaceflight hardware results in alterations to the transcriptome and proteome. Life Sci. Space Res. *15*, 88–96.

55. Herranz, R., Vandenbrink, J.P., Villacampa, A., Manzano, A., Poehlman, W.L., Feltus, F.A., Kiss, J.Z., and Medina, F.J. (2019). RNAseq analysis of the response of Arabidopsis thaliana to Fractional gravity under blue-light Stimulation during spaceflight. Front. Plant Sci. *10*, https://doi.org/10.3389/fpls.2019.01529.

56. Vandenbrink, J.P., Herranz, R., Poehlman, W.L., Alex Feltus, F., Villacampa, A., Ciska, M., Javier Medina, F., and Kiss, J.Z. (2019). RNA-seq analyses of Arabidopsis thaliana seedlings after exposure to blue-light phototropic stimuli in microgravity. Am. J. Bot. *106*, 1466–1476.

57. Manzano, A., Creus, E., Tomás, A., Valbuena, M.A., Villacampa, A., Ciska, M., Edelmann, R.E., Kiss, J.Z., Medina, F.J., and Herranz, R. (2020). The

FixBox: hardware to provide on-orbit fixation capabilities to the EMCS on the ISS. Microgravity Sci. Technol. *32*, 1105–1120.

58. Manzano A., Villacampa A., Sáez-Vásquez J., Kiss J.Z., Javier Medina F., Herranz R.. The importance of Earth reference controls in spaceflight –omics research: characterization of nucleolin mutants from the Seedling Growth experiments. iScience. doi:10.2139/ssrn.3661944

59. Herranz, R., Anken, R., Boonstra, J., Braun, M., Christianen, P.C., de Geest, M., Hauslage, J., Hilbig, R., Hill, R.J., Lebert, M., et al. (2013). Ground-based facilities for simulation of microgravity: organism-specific recommendations for their use, and recommended terminology. Astrobiology *13*, 1–17.

60. Polo S.-H.L., Saravia-Butler A.M., Boyko V., Dinh M.T., Chen Y.-C., Fogle H., Reinsch S.S., Ray S., Chakravarty K., Marcu O., et al RNAseq analysis of rodent spaceflight experiments is confounded by sample collection techniques.bioRxiv doi:10.1101/2020.07.18.209775

61. Kruse, C.P.S., Basu, P., Luesse, D.R., and Wyatt, S.E. (2017). Transcriptome and proteome responses in RNAlater preserved tissue of Arabidopsis thaliana. PLoS One *12*, e0175943.

62. Choi, W.-G., Barker, R.J., Kim, S.-H., Swanson, S.J., and Gilroy, S. (2019). Variation in the transcriptome of different ecotypes of Arabidopsis thaliana reveals signatures of oxidative stress in plant responses to spaceflight. Am. J. Bot. *106*, 123–136.

63. Ray, S., Gebre, S., Fogle, H., Berrios, D.C., Tran, P.B., Galazka, J.M., and Costes, S.V. (2019). GeneLab: omics database for spaceflight experiments. Bioinformatics *35*, 1753–1759.

64. Berrios, D.C., Beheshti, A., and Costes, S.V. (2018). FAIRness and usability for open-access omics data systems. AMIA Annu. Symp. Proc. *2018*, 232–241.

65. Wilkinson, M.D., Dumontier, M., Aalbersberg, I.J., Appleton, G., Axton, M., Baak, A., Blomberg, N., Boiten, J.W., da Silva Santos, L.B., Bourne, P.E., et al. (2016). The FAIR Guiding Principles for scientific data management and stewardship. Sci. Data *3*, 160018.

66. Berrios, D., Weitz, E., Grigorev, K., Costes, S., Gebre, S., and Beheshti, A. (2020). Visualizing Omics Data from Spaceflight Samples Using the NASA GeneLab Platform. In Proceedings of the 12th International Conference on Bioinformatics and Computational Biology, *70*, Q. Ding, O. Eulenstein, and H. Al-Mubaid, eds., pp. 89–98, https://doi.org/10.29007/rh7n.

67. Barker, R., Lombardino, J., Rasmussen, K., and Gilroy, S. (2020). Test of space transcriptome: a discovery environment to explore multiple plant biology spaceflight experiments. Front. Plant Sci. *11*, 147.

68. Beheshti, A., Miller, J., Kidane, Y., Berrios, D., Gebre, S.G., and Costes, S.V. (2018). NASA GeneLab project: bridging space radiation omics with ground studies. Radiat. Res. *189*, 553–559.

69. Haendel, M.A., Chute, C.G., and Robinson, P.N. (2018). Classification, ontology, and precision medicine. N. Engl. J. Med. *379*, 1452–1462.

70. Yachie, N., Robotic Biology Consortium, and Natsume, T. (2017). Robotic crowd biology with Maholo LabDroids. Nat. Biotechnol. *35*, 310–312.

71. Shaghaghi, A., and Antonakopoulos, K. (2012). The societal impacts of a mars mission in the future of space exploration. Phys. Proced. 176–185, https://doi.org/10.1016/j.phpro.2012.08.021.

72. Han, X., Wang, R., Zhou, Y., Fei, L., Sun, H., Lai, S., Saadatpour, A., Zhou, Z., Chen, H., Ye, F., et al. (2018). Mapping the mouse cell atlas by microwell-seq. Cell *173*, 1307.

73. Regev, A., Teichmann, S.A., Lander, E.S., Amit, I., Benoist, C., Birney, E., et al. (2017). How to build a human cell atlas. Nature *547*, 24, https://doi.org/10.7554/eLife.27041.

74. Schmidt, M.A., Schmidt, C.M., Hubbard, R.M., and Mason, C.E. (2020). Why personalized medicine is the frontier of medicine and performance for humans in space. New Space, 63–76, https://doi.org/10.1089/space.2019.0037.

75. Schmidt, M.A., and Goodwin, T.J. (2013). Personalized medicine in human space flight: using Omics based analyses to develop individualized countermeasures that enhance astronaut safety and performance. Metabolomics *9*, 1134–1156.

76. Stingl, J.C., Welker, S., Hartmann, G., Damann, V., and Gerzer, R. (2015). Where failure is not an option –personalized medicine in astronauts. PLOS ONE, e0140764, https://doi.org/10.1371/journal.pone.0140764.

77. Zwart, S., Gibson, C., Mader, T., Ericson, K., Ploutz-Snyder, R., and Smith, S. (2012). Vision changes after spaceflight are related to alterations in folate– and vitamin B-12–dependent one-carbon metabolism. SciVee. https://doi.org/10.4016/38821.01.

78. Zwart, S.R., Gregory, J.F., Zeisel, S.H., Gibson, C.R., Mader, T.H., Kinchen, J.M., Ueland, P.M., Ploutz-Snyder, R., Heer, M.A., and Smith, S.M. (2016). Genotype, B-vitamin status, and androgens affect spaceflight-induced ophthalmic changes. FASEB J. *30*, 141–148.

About the Organization

The International Standards for Space Omics Processing (ISSOP) is an international consortium of scientists who recognize the growing need for sample-processing standardization and metadata normalization of space omics studies at the global level. It was created early in 2020 with a balanced international scope with perspectives from multiple space communities from Japan (including Living in Space, Omics Subgroup), Europe (including Space Omics Topical Team), and the United States (including GeneLab Analysis Working Group). ISSOP plans to develop standardized science for these data to enhance the robustness of the field and potentially launch an upcoming era of human space omics.

PATTER, Volume *1*

Supplemental Information

A New Era for Space Life Science: International Standards for Space Omics Processing

Lindsay Rutter, Richard Barker, Daniela Bezdan, Henry Cope, Sylvain V. Costes, Lovorka Degoricija, Kathleen M. Fisch, Mariano I. Gabitto, Samrawit Gebre, Stefania Giacomello, Simon Gilroy, Stefan J. Green, Christopher E. Mason, Sigrid S. Reinsch, Nathaniel J. Szewczyk, Deanne M. Taylor, Jonathan M. Galazka, Raul Herranz, and Masafumi Muratani

Figure S1. Growth of GeneLab omics datasets by release year

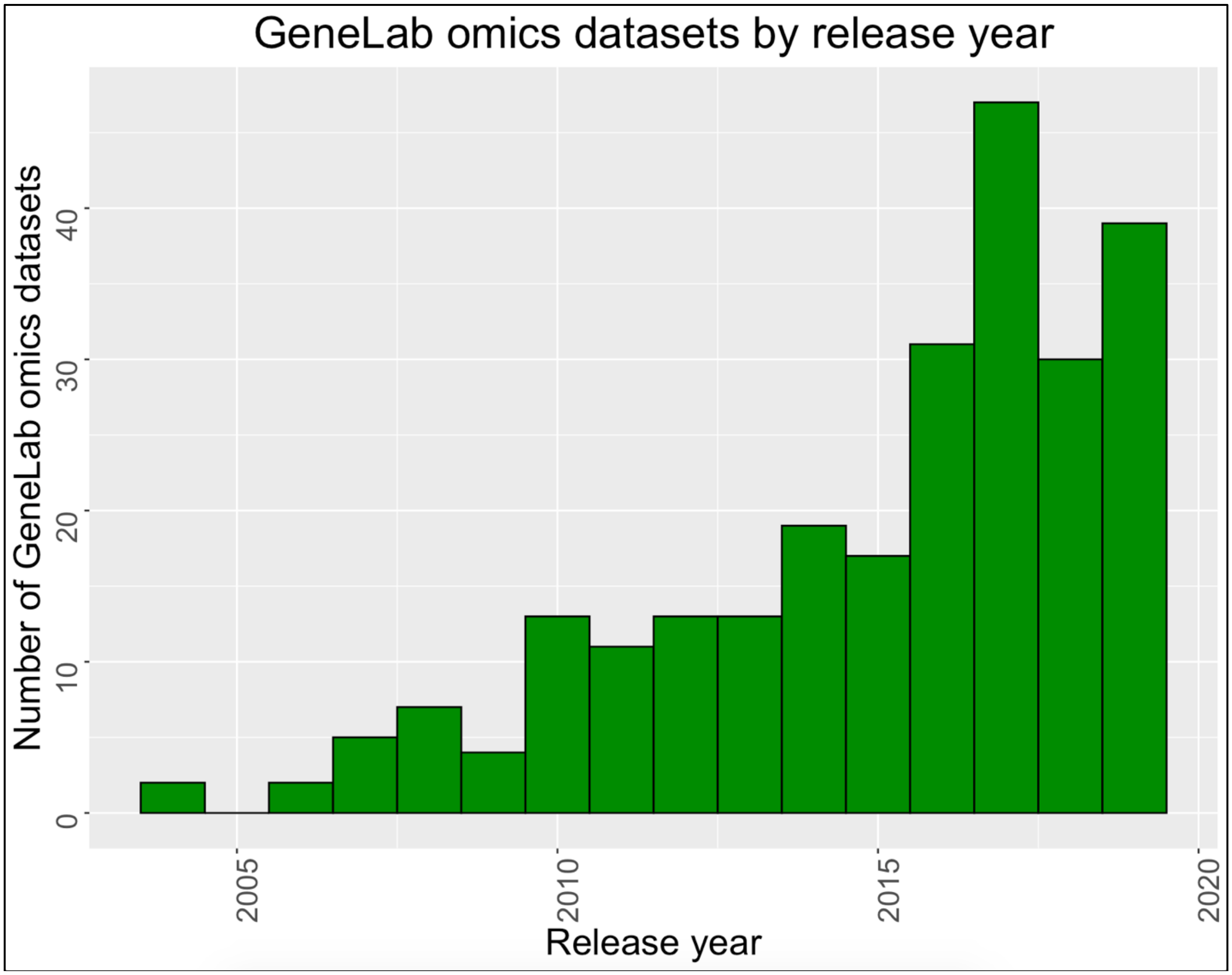

Figure S1. Growth of GeneLab omics datasets by release year

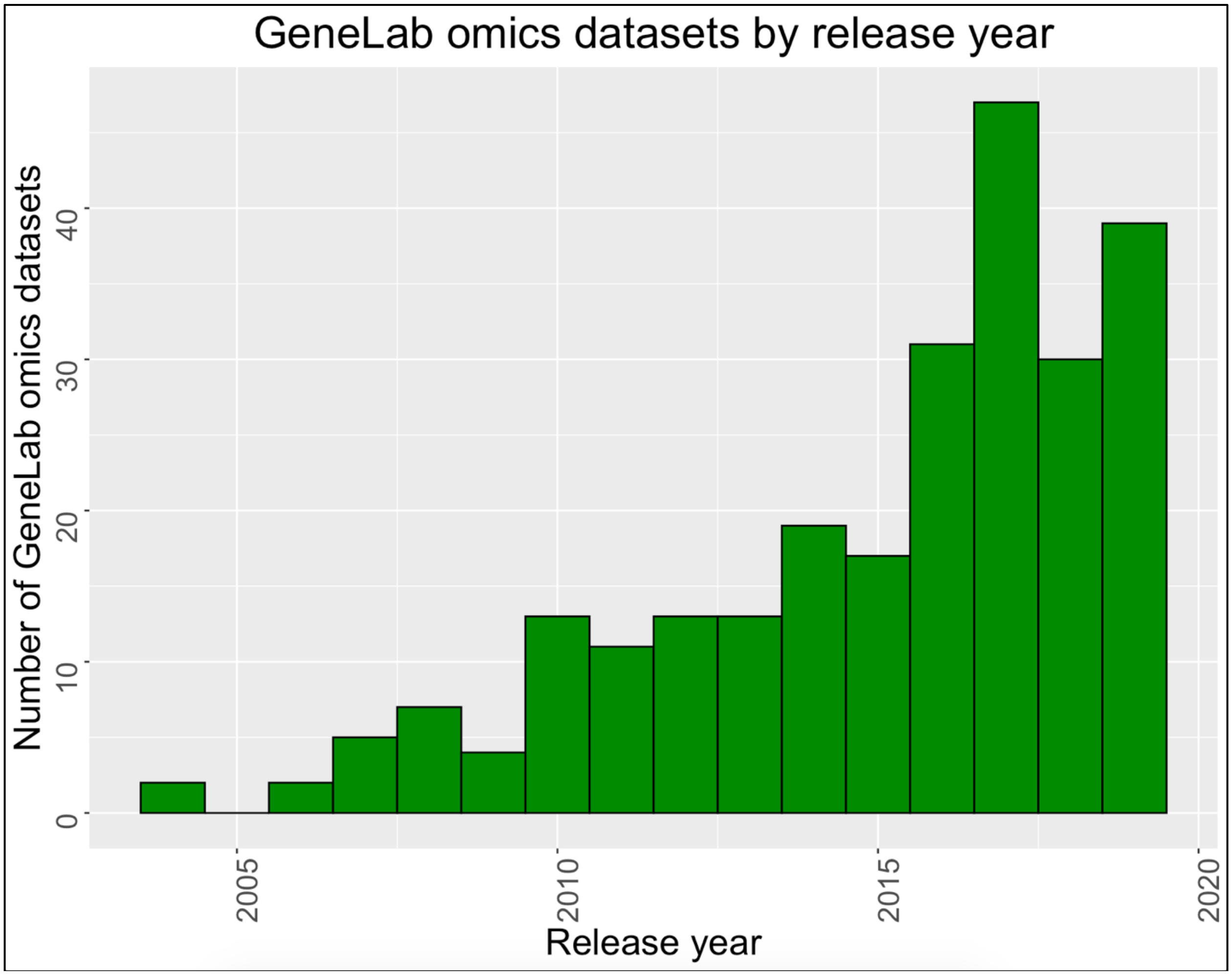

Supplement: Document S2. Article plus Supplemental Information [file mmc2.pdf]
